# What are the challenges for implementing an “organic label” to camel milk?

**DOI:** 10.3389/fnut.2023.1288553

**Published:** 2023-11-15

**Authors:** Gaukhar Konuspayeva, Bernard Faye, Moldir Nurseitova, Shynar Akhmetsadykova

**Affiliations:** ^1^Department of Biotechnology, Al-Farabi Kazakh National University, Almaty, Kazakhstan; ^2^Research and Production Enterprise “ANTIGEN” Co., Ltd., Almaty, Kazakhstan; ^3^UMR SELMET, International Campus of Baillarguet, CIRAD-ES, Montpellier, France; ^4^LLP Kazakh Research Institute for Livestock and Fodder Production, Almaty, Kazakhstan

**Keywords:** organic milk, camel, contamination, residues, standard, label

## Abstract

Increasing demand for camel’s milk worldwide occurred in the context of the development of the organic sector in agriculture. The implementation of an organic label for camel milk has never been established. However, the creation of such a label faces to important challenges that are investigated in the present paper. Indeed, although camel milk conveys the image of a “natural product” issued from remote places, the risk of being produced in contaminated areas (mining activities, oil extraction) cannot be neglected for grazing animals. Moreover, the management of veterinary drugs for prevention or curative treatment can lead to the presence of residues in milk, especially in camel species with different pharmacokinetics, although similar instructions than for cow milk are used. Moreover, the lack of international standards regarding both composition and hygienic rules, the risks of adulteration, and the necessity to use specific indicators or analytical procedures adapted to the behavior of camel milk, have to be taken in account in the establishment of the specifications for the camel milk producers through the world.

## 1. Introduction

Camel milk production worldwide has been steadily increasing in recent decades, with relative proportions greater than those of all other dairy species (1). With an estimated production of 3,114 million tons in 2021 (2), widely underestimated, camel milk is available in much smaller quantities than for other dairy species, i.e., 0.34% of the world’s milk production available on the market. However, the annual growth since 1961 (first available data in FAOstat database) is estimated to 6.5%, which is much higher than cow (2.3%), goat (3.2%), and sheep milk (1.7%); only buffalo milk showed more important change, with an annual growth of 11.0%.

Obviously, such an increase in camel milk production must be related to growing demand, especially the onset of the product “camel milk” on the market (3). For a long time, camel milk produced by animals under extensive systems managed by pastoralists was mainly reserved for household consumption (4, 5). The introduction of camel milk to the market has led to the emergence of more intensive production systems and is therefore more dependent on food or veterinary inputs (6). Such a change in the way of producing the camel milk “from the dune to the barn” (7) has obviously a potential impact on the status of the product. Formerly regarded as a “natural product” obtained in low-input production systems, camel milk produced in “modernized farms” is nowadays confronted with similar challenges as milk from other dairy species. In this context, the growing demand for organic milk worldwide (8) has led to camel farming.

There are many explanations and definitions for organic agriculture but according to FAO definition: “all converge to state that it is a system that relies on ecosystem management rather than external agricultural inputs.” It is a system that considers potential environmental and social impacts by eliminating the use of synthetic inputs, such as synthetic fertilizers and pesticides, veterinary drugs, genetically modified seeds and breeds, preservatives, additives, and irradiation. These practices have been replaced by site-specific management practices that maintain and increase long-term soil fertility and prevent pests and diseases. Regarding camel farming systems, many approaches to water, soil, and feeding/fodder are applicable to other organic dairy animals. However, camels are classified as pseudo-ruminants and that they lack the gallbladder. This makes the camel physiology (nutrition, reproduction, lactation) quite different notably with respect to veterinary drug exposure and withdrawal times. This is especially true for the evolution of the pharmacokinetics of drugs in camel milk and meat. Currently, limited data are available on the specificity of camel treatment and the method of prevention at camel farms to avoid classical veterinarian drugs. Moreover, references regarding the criteria to establish an organic status specifically to camel milk is completely lacking.

Thus, the main objective of the present paper was to assess the main challenges for camel milk producers and processors to get an “organic label” according to the current knowledge on this product and its conditions of production and processing. In such context, it is necessary to review a “state-of-art” regarding the status of (i) contamination (heavy metals, POPs, medicine residues), (ii) hygienic conditions or adulteration control, and (iii) physico-chemical and microbiological parameters standard of raw and processed milk.

## 2. The camel milk, a “natural product”?

Camel milk is usually presented by sellers as “the white gold of the desert,” with animals fed on natural pastures in arid ranges (9). From vast semi-desert spaces, far from polluting industries and urban concentrations, and from intensive agricultural areas, camel milk easily conveys the image of a product close to an intact nature and devoid of deleterious elements. However, this image is otherwise misleading or at least not taking into account the risks of contamination, and has been undermined here. Studies and work have been carried out in different countries, notably regarding the risks of environmental contaminations. Obviously, these risks are not different than for milk from other species (cow, sheep, goat, buffalo). Moreover, camel milk produced in good conditions in a safe environment is beneficial for the consumers, but get an organic label, it is important to have a clear assessment of the potential risks.

### 2.1. Contamination by heavy metals

Heavy metals have been found in milk collected from camels living close to mining areas or oil extraction zones. Thus, in Iran, although the values were below the European standard (EU rule n° 2021/1323 on 10/08/2021), lead, cadmium, nickel, and chromium were detected in camel milk from different regions with mean concentrations of 4.46 ± 0.65 μg/L, 0.30 ± 0.05 μg/L, 0.53 ± 0.02 μg/L, and 0.03 ± 0.00 μg/L, respectively (10). Conversely, in Saudi Arabia, Elamin and Wilcox (11) reported a considerable value of lead in some samples of camel milk (180 μg/L, i.e., 9 times the limit value of 20 μg/L). Moreover, in Saudi Arabia, in the Western region, Elhardallou and El-Naggar (12) detected high concentrations of cadmium in camel milk, between 7 and 89 μg/L, that were confirmed later as sites close to the oil extraction industry (13). Chromium and strontium have also been detected in camel milk. In polluted steppic areas of Kazakhstan, Konuspayeva et al. (14) reported lead concentration above the accepted limit (25 ± 19 μg/L). In another study, a high value of lead (30 μg/L) was observed again, whereas 2 μg/L of cadmium was lower than the acceptable limit (5 μg/L) (15). More recently, heavy metals have been detected in camel milk in Nigeria (16, 17), Iran (18), Kenya (19), and Pakistan (20).

However, one important challenge to assess the contamination status of milk is the reliability of the reported values to compare with the expected standard. For example, in a study on the presence of heavy metals in camel milk in Kazakhstan, several samples were analyzed by three different laboratories: two in Kazakhstan using conventional methods available in the country and the other in France based on standard methods with reference samples (commercially available, such as AccuTraceTM Reference Standard solutions). Laboratory 1 (Kazakhstan) used an ICP apparatus, Laboratory 2 (Kazakhstan) used a volt-amperometric method, and Laboratory 3 (France) used an atomic absorption spectrophotometer. Thus, the content values for conventional minerals such as zinc varied from 0.4 to 4.9 ppm, for iron from 0.46 to 1.72 ppm, and for copper from 0.09 to 0.51 ppm. For pollutants such as lead and arsenic, variations of a factor of five were also observed (21). Such discrepancies between laboratories underline the importance of harmonization of methods in camel milk producing countries, especially to assess the conformity of the milk samples with the specifications for organic milk.

### 2.2. Contamination by persistent organic pollutants (POPs)

Monitoring the levels of highly toxic POPs and pesticides with high bioaccumulation abilities in the food chain, which cause serious health challenges, is still uncommon in developing countries (22–24).

The main cause of camel milk contamination by different hazardous pesticides is the presence of their residues in animal feedstuffs because of the use of banned substances to control plant pests, pesticide application to farm animals, environmental contamination, and accidental spills (15, 24–26). Al Ali et al. (27) reported cases of previously healthy farmers who presented with symptoms of cholinergic crisis accompanied by slurred speech, headache, vomiting, diarrhea, frequent micturition, muscle fasciculation, chest discomfort, atrial fibrillation, and bradycardia that developed several hours after milk ingestion from the mammary glands of camels treated with organophosphate pesticides (OPPs) against the mite *Sarcoptes scabiei cameli*, agent of mange, one of the most common diseases in camel farms (28).

Traces of hexachlorocyclohexane (HCH), lindane, and hexanchlorothalonil were detected in camel milk and fermented camel milk (shubat) sampled from different regions of Kazakhstan (29). Traces of various pesticides were determined in camel milk samples mostly from southern regions with intensive agricultural crop production, showing their presence but in concentrations under European thresholds. However, the concentration of dioxine (DL-PCB) in samples from the petrol extraction region exceeded the European Standards. The results seem to indicate a link between contamination of plants and that of milk for two pesticides, γ-HCH and 4,4-DDD, an isomer of Dichlorodiphenyltrichloroethane-DDT (15).

Organochlorine pesticides (OCPs) can be present in various environmental and food matrices for long periods after their use and trade (30, 31). According to (32), the content of chlorinated pesticides in cow milk varies according to the lactation phase, sanitary condition of the udder, age of the animal, and number of gestations. The milk sampling season can also affect pesticide concentrations (33). Wong and Lee (34) showed that certain OCPs (including DDT and lindane) could have contaminated livestock milk 10 years after their prohibition. Al-Hawadi et al. (30) investigated the extent of pesticide contamination in camel milk, meat, and liver in Jordan. As a result, 31.7% of the examined milk, was contaminated with OCPs, 20% of camel milk samples were found positive and had concentrations higher than Maximum Residue Limit (MRL) for DDTs (0.067 mg/kg fat in milk); HCHs isomers (α, β, and γ) have been detected in 13% of camel milk samples, γ-isomer (lindane), has been found with a mean concentration of 0.260 mg/kg fat, exceeding its MRL value (0.01 mg/kg fat); heptachlor has been detected with a mean concentration of 0.07 mg/kg fat, exceeding its MRL (0.006 mg/kg fat); dieldrin has been detected with a mean concentration of 0.020 mg/kg fat.

Wanjiku et al. (31) determined OCP and OPP residues in camel milk in Kenya. The mean heptachlor concentration in several camel milk samples was above the MRLs of the EU Pesticides Database (EUPD MRLs) and the MRLs set by the *Codex Alimentarius*. In contrast, the detected concentrations of OCPs were low compared to the MRLs established by the *Codex Alimentarius*, EUPD, and the United States Department of Agriculture. Furthermore, up to 70% of camel milk samples were contaminated with malathion. In Pakistan, during the monitoring of pesticide traces in different types of milk, among the camel milk samples, bifenthrin, chlorpyrifos, carbofuran, deltamethrin, imidacloprid, and lambda-cyhalothrin were detected (26).

Thus, similarly to heavy metals, the contamination of camel milk by pollutants can occur in grazing animals. The attribution of one label “organic” to camel milk, especially for producers having their animals grazing next to potential sources of pollution (such as oil producing areas, or conventional cotton field), could be highly problematic.

### 2.3. Contamination by medicines

In pastoral areas, access to veterinary medicine inputs is often difficult (35) either because veterinary pharmacies are absent or because hawkers sell adulterated products. Moreover, often left to their own devices, pastoralists hardly respect prescribed doses. Thus, the presence of medicinal residues in milk (antibiotics, pest control products, anthelmintics, and anti-inflammatory agents), although rarely explored in camel milk, is likely frequent. Investigations on the presence of sulfadimidine (36), oxytetracycline (37), anthelmintics (38) or dexamethasone (39) have been conducted, but little data are available on the pharmacodynamics of most of the medicines used in camels, despite the specificity in the metabolism of these molecules in this animal (40). For example, the rules used for discarding milk after antibiotic treatment are based on data collected from cow milk, without considering the specificity of camel milk (41). Notably, the pharmacokinetics of marbofloxacin in camels are characterized by a higher maximum plasma concentration and area under the plasma concentration-time curve, more rapid absorption following IM administration, and longer terminal half-life than in cows (42). Further studies should be required to assess the importance of medicine residues in camel milk and overall, to specify the withdrawal period for this species known for its slower pharmacokinetic and metabolism (43).

## 3. A standard for camel milk

Camel milk, similar to all products intended for human consumption, is subject to market rules. The recent introduction of camel milk into the market has pushed the need for camel milk-producing countries to comply with international quality and safety standards. These standards are classically divided into microbiological (safety) and physicochemical (quality) standards. Cow’s milk, which accounts for more than 82% of the milk consumed worldwide, is often the norm for other milks, especially “marginal” ones such as camel milk, although the physicochemical composition (44) and the response capabilities to bacterial contamination can be different (45). The establishment of a standard for camel milk must include two major elements: (i) to be sure that marketed milk does not pose any health problems to consumers (food safety standard), and (ii) to be sure that the product sold corresponds to what has been produced both in terms of physicochemical composition and nutritional value; in other words, no “deception” on the goods is present.

In most countries that produce camel milk, national standards are available for the dairy industries that process camel milk. These standards are not always published, discussed, or implemented, particularly because they are based on regulations concerning cow milk. In general, they include specifications for the structures, machinery, cleaning systems, packaging devices, and packaging used for cow milk. However, not all these elements are applicable to camel milk. At the international level, the problem is accentuated by the fact that norms and standards are most often imposed by industrialized countries that are high producers and exporters of dairy products (USA, Australia, European Union, etc.) that do not have developed dairy camel farming, or where the camel does not appear clearly as a dairy-producing animal despite the recent implementation of camel dairy farms in Europe and the USA (46). In this respect, camels have been absent for a long time from the list of animals producing milk intended for human consumption in the *Codex Alimentarius*.

For countries in Central Asia, the national standards already used in the Soviet Union (USSR) were available. Since 1986, these standards have been used for camel milk and *shubat*: RST KazSSR 166-86 and RST KazSSR 117–91 (47). Furthermore, since the independence of Kazakhstan in 1991, 15 standards for camel milk and their derivatives have been established. Following the creation and establishment of the Eurasian Economic Customer Union, technical regulations of the EU were developed in 2011–2013, and these regulations have been superior to national standards since 2015 (48, 49). The last national standard in Kazakhstan has lost its official status despite updated and improved versions of its physicochemical and other characteristics.

However, some contradictions were systematically observed. Pasteurized camel milk must be used for fermented camel milk called *shubat* in Kazakhstan. However, there are no data or standards regarding the pasteurization procedure (time, t°) or a global standard for pasteurized camel milk. To produce camel milk powder in 2015, the standard for raw materials was described in pasteurized form. Currently, a document describing the raw matter supposed to be processed into fermented or powdered milk exists but between raw milk and fermented or powdered milk, the step of “pasteurization is lacking (23).

Thus, because the establishment of a standard for organic milk must be able to rely on an available standard for conventional raw or pasteurized milk, the lack of international standard, or the diversity of national ones when they exist, also represents an important challenge to set up convenient specifications. Even if the camel milk is produced in safe pastoral areas, it could get easily a specific label as “produced in natural pasture,” but it is not sufficient to state that it is an “organic product.” Similar difference is observed in fish sector where a label “sustainable fish production” is possible to get for wild fish collection while organic status can be attributed to fish farms respecting organic specifications (50).

### 3.1. Hygienic quality standard

The sanitary quality of milk depends on the conditions of production: hygienic milking conditions and management practices such as feeding and prophylaxis. Contrary to certain beliefs, camel milk can spoil rapidly during storage and transport. The belief that high quantities of proteins with antibacterial properties, such as lactoferrin, lysozyme, lactoperoxidase, and immunoglobulins, could have a beneficial effect on their preservation at ambient temperature is questionable (51), and the coliform develops, which is a rapid and continuous process. As a result, camel milk still faces various problems, such as high postharvest quantity losses up to 50%, quality deterioration due to poor staff hygiene, inadequate cleaning of production lines, and lack of knowledge and skills for camel health management (52).

Assessment of the bacterial load in milk is a criterion widely used worldwide to qualify the hygienic status of cow, sheep, and goat milk collected from farm dairies. The generally accepted standards are less than 100,000 UFC/ml (10^5^) of total flora for delivering raw milk intended for consumption (category A milk), and less than 300,000 UFC/ml (3 × 10^5^) for milk intended for pasteurization. In most surveys, these values are higher than the standards (53–56).

Regarding the pathogens, the potential for contamination of camel milk is the same as for other milks, and the standard for good-quality milk followed by cow milk is the absence of Salmonella and other pathogens, less than 100 CFU/mL. It should be noted that camel milk requires testing of all milk samples for each process by the same assessment as cow milk quality: methylene blue test, resazurin test, boiling test, etc. (57). According to different authors, the main pathogens isolated from camel milk were *Streptococcus*, *Staphylococcus* sp., *Corynebacterium*, *Bacillus*, and *Escherichia* (58, 59). Moreover, most of the isolated camel milk pathogens show resistance to antibiotics (tetracycline, ampicillin, methicillin, cefoxitin, streptomycin, and cefaclor) (57, 59).

However, the standard for food safety is based on the specificities of cow milk without taking in account those of camel milk. For example, in the absence of use of silages for camel feeding in most of the cases, the risk of presence of listeria is very low or even zero (60). Yet, the determination of *Listeria* in camel milk is systematic to get certification of healthy milk without clear risk assessment.

The somatic cell count (SCC) is also an acceptable standard for raw cow milk, with values not exceeding 120,000 cells according to the European standards for milk to be classified as Category A. These values depend on the physiology of the mammary glands and milk synthesis. For small ruminants, the border values differed from those for cattle. Few studies where SCC is described for camel milk, are available (61, 62). Therefore, it is necessary to establish a definitive acceptable limit for a proper standard for camel milk (63).

### 3.2. Physico-chemical composition

**Figure 1:**
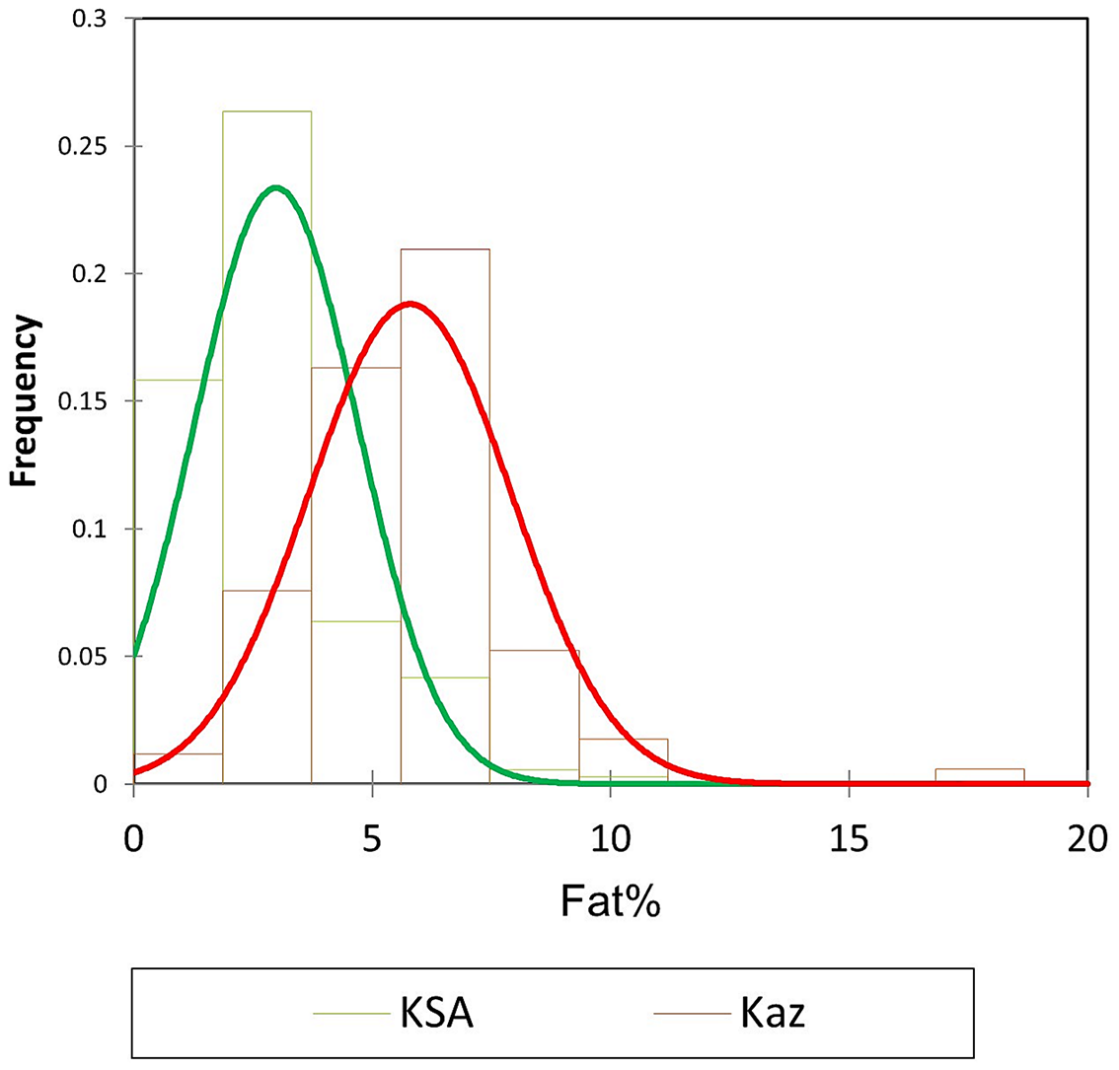
Distribution of fat percentage values in two monitoring of individual camel milk compositions in Saudi Arabia (KSA in green) and Kazakhstan (Kaz in red).

Analytical methods used to investigate the physicochemical properties are currently standardized, and the methods used for cow milk and other species can be used for camel milk. However, the observed variability in the different components of camel’s milk can be a constraint for establishing a convenient standard like for standard of food safety discussed above. Two examples are presented here:

- Regarding physical properties, a recent study (64) investigated the use of freezing point as an indicator in the dairy industry to detect milk adulteration (for example, adding water). Need to underline that until now reference value for freezing points for “normal” camel milk is not defined by reference method. Only descriptions exist in the few published data on camel milk in the literature, including only routine analytical methods such as automatic milk analyzers. Generally, the calibration of the apparatus for camel milk was not verified. Such monitoring, without establishing a reference value, does not provide any information or interest. However, Konuspayeva, et al. (64), after using thousands of samples over several months, analysis with the reference method (by cryoscope) and express method (by automatic analyzer) showed a weak correlation between these two methods and quite different values. Based on 680 milk samples for which the reference method was determined, the mean value was −0.332 ± 0.030°C (range − 0.260 to −0.478). No data are available regarding the freezing point of camel milk adulterated with water, and this shows the necessity, at least, to calibrate the devices used in the dairy industry for camel milk. The cost of camel milk is higher than that of cow milk; consequently, the risk of adulteration of camel milk is higher. Therefore, establishing a reference value for the freezing point of camel’s milk is important.
- Regarding milk composition, an important variation factor (notably for fat content) beyond the lactation stage is the quality of milking (manual or machine). Indeed, in camels, the proportion of cisternal milk is around 5–15% only vs. more than 35% in dairy cows, requiring a stronger simulation to obtain the remaining alveolar milk, usually richer in fat (65). As camel milk is processed (fermented milk, pasteurized milk, powdered milk, cheese, and other products), the standardization of processes is also an important issue from the perspective of industrialization of the camel milk sector (23). Moreover, important geographical variability was observed, including differences between breeds and species of large camelids (dromedary or Bactrian), as shown by the comparative study of Faye et al. (66). For example, the values of fat percentage in two different individual monitoring of camel milk composition in Kazakhstan (44) and in Saudi Arabia (67) appeared on average significantly different (*p* < 0.001) with a mean of 2.98 ± 1.70% in Saudi Arabia and 5.79 ± 2.1% in Kazakhstan (Figure 1). Obviously, the variability in camel milk components is not necessarily important to get an organic status, but such geographical variability underlines the difficulties to get international standard. More generally, in a meta-analysis carried out on a large sample of references relating to the composition of camel milk, Al-Haj et al. (68) clearly showed on the one hand the significant heterogeneity of the published results and on the other hand, the numerous biases observed in the selection of samples, the classification of interventions, missing data, analytical methods or the selection of results.

### 3.3. Processing indicators

The examples cited above regarding milk composition emphasize the importance of harmonizing methods and applying them to international standards. Standardization of methods must also include the processes used for making dairy products and controlling milk specificity. For example, in the case of pasteurization, specific conditions and indicators must be determined. Although there is no difference in the establishment of an organic label for pasteurized camel milk compared to conventional one on this topic, it is important to recall here that alkaline phosphatase (ALP), traditionally used to determine the success of cow milk pasteurization, is not a convenient indicator in camel milk because the heat resistance of camel ALP, firstly reported by Loiseau et al. (69) and later by Wernery et al. (70). Loiseau et al. (69) suggested using glutamyl transpeptidase (GGT) or leucine arylamidase as indicators of the pasteurization of camel milk. Later, Lorenzen et al. (71) reported that lactoperoxidase (LPO) was a more appropriate indicator of pasteurization. However, currently, there have been no sufficiently in-depth studies in this field, even though pasteurized camel milk has been introduced into the international market. This example shows the importance to take in account the specificity of camel milk in the assessment of its adequacy with the international regulations. The establishment of an organic label for such milk cannot be based exactly on the same criteria than for cow milk.

### 3.4. Adulteration control

Regarding the specificity of camel milk, the higher the cost of products, the more likely it is to be mixed with milk from other species. Some findings have been made but are not available for a few industries. This point is important because of the difference in price between camel milk, which is usually more expensive, and other milks (3). Several methods are available to check the specificity of milk, including testing a mixture of goat and cow milks for immunoglobulins. The peculiarity of camel milk is its absence of β-lactoglobulin, so its presence in milk can be considered a marker of fraud, as has been used in Iran (72).

A commercial test using the same peculiarity as camel milk, based on the radial immunodiffusion (RID) reaction (technique applying precipitation antigen–antibody system), was proposed in France based on specific antiserum against the bovine, caprine, or ovine protein to be measured. The diameter of the precipitate halo was directly proportional to the protein concentration in the sample. The duration of the test ranged from 4 to 6 h after the start-up, which made it possible to interpret the results during the day. It is a very sensitive test because it can detect the presence of only 0.1% cow, goat, or sheep whey in camel whey or in milk diluted to 1/2 or 1/3, with detection limits of 0.2 and 0.3%, respectively. In China, governmental control of adulteration is achieved mainly by PCR, which takes 48 h and is costly and time-consuming.

The adulteration of milk can also involve the introduction of vegetal oil in substitution to milk fat, leading to a change in fatty acid composition and sterols (73). The establishment of an organic label is not compatible with such adulteration which must be correctly detected by convenient method, notably the determination of the presence of phytosterols in the milk (73).

## 4. The challenges to get an organic camel milk

From the previous observations, there are three main challenges to obtaining an organic label for camel milk.

- The absence of “normal” standard (non-organic) camel milk at the international level. The history of camel milk standards in Kazakhstan and the Eurasian Customer Union has been mentioned above, but unfortunately, it is not the same in other parts of the world. In the Middle East and in North African countries, the establishment of standards is either underway or under development. In sub-Saharan countries, the development of such a standard is not even initiated. Due to the lack of international standards, different regional standards must be proposed.
- Limited data are available on the use of veterinary drugs in camels and their residues in milk. The use of possible alternative products is currently lacking. Until now, most veterinary drug discoveries have not mentioned the conditions of specific applications for lactating camels. Moreover, for the use of drugs in veterinary practice, the camel is included as a global entity under the term “ruminant,” despite the specificity of its pharmacokinetic, as mentioned above: higher plasma concentration, faster absorption following administration, longer elimination time compared to cows (37–40).
- In pastoral systems, the camel diet is highly diversified (74) and based on a widely dispersed resource, examining the potential contamination by heavy metals or POPs is difficult (75). In contrast, in intensive systems based on feeding irrigated fodders and concentrates, specifications similar to those used in cattle breeding can be applied. In other words, it is not necessarily easier to obtain organic camel milk labels in extensive areas than in intensive areas, especially for veterinary drugs, as mentioned above.

## 5. Conclusion

The development of a label “organic camel milk” would be largely appreciated by the consumers who have, generally, a positive image of the camel and its products regarded as “natural” or “health-benefit.” Camels living in remote arid zones provide a better opportunity to valorize their production. However, the establishment of the label “organic camel milk” faces important challenges, as it has been showed in the present paper. The way to get such label could be a bumpy way because the risks of contamination of soils, pastures, and water in arid zones have to be taken in consideration although most of the camel farming systems is linked to expected “natural environment.” Moreover, further studies have to be achieved to make clearer the deadlines for withdrawal of products (milk, meat) in the event of use of medicinal products, in particular antibiotics. In addition, better management practices regarding the use of medicine must be promoted among the camel breeders. No reference existing on organic camel milk, it is expected that the present “state-of-art” could contribute to the establishment of the right criteria to get this status.

## Author contributions

GK: Conceptualization, Funding acquisition, Supervision, Validation, Writing – review & editing. BF: Writing – original draft. MN: Writing – review & editing. SA: Data curation, Writing – review & editing.
